# Investigation of the Anthropometric Changes in Breast Volume and Measurements After Breast Reduction

**DOI:** 10.7759/cureus.4312

**Published:** 2019-03-25

**Authors:** Ümran Muslu, Emre Demir, Fikri Özdemir, Vahdet Özkoçak, Engin Yıldırım

**Affiliations:** 1 Plastic Surgery, Hitit University Faculty of Medicine, Çorum, TUR; 2 Biostatistics, Hitit University Faculty of Medicine, Çorum, TUR; 3 Anatomy, Hitit University Faculty of Medicine, Çorum, TUR; 4 Anthropology, Hitit University Faculty of Science and Literature, Çorum, TUR; 5 Obstetrics and Gynecology, Hitit University Faculty of Medicine, Çorum, TUR

**Keywords:** breast reduction, breast volume, anthropometric methods

## Abstract

Objective

This study aims to compare breast volume changes and other anthropometric measurements by using before and after breast reduction pictures of women who underwent breast reduction operation in Plastic and Reconstructive Surgery clinic and by performing measurements from the anatomic points indicated in the literature.

Background

Landmarks (previously identified as anatomic points) that show the success of breast reduction operation are not sufficient. Anthropometric points and their identification are of great importance for choosing the landmarks and identifying the statistical approaches to be used.

Methods

A total of 40 women were measured breast anthropometric measurements in pre- and post-operative breast reduction surgery changes by a photographic technique using Image J programme from the anatomical points determined in the literature. Comparison of right and left breast anthropometric measurements before and after the operation was performed using the paired t test or Wilcoxon signed rank test. The intraclass correlation coefficient (ICC) and Bland-Altman plots were used to determine the agreement between each pair of measurements.

Results

There was a statistically significant agreement between all the measurements (p<0.001). According to the Bland-Altman graphics, right and left breast measurements after the operation were within the limits of agreement according to all measurement points.

Conclusion

This study presented anthropometric measurements to show and guide patient satisfaction and aesthetic success of the operations performed by plastic surgeons.

## Introduction

There is a dramatic improvement in patients’ physical and psychological symptoms after breast reduction. Patients become more positive about life and the future when they finally have healthy and aesthetic breasts. Identifying the ideal measurements and reconstructing these measurements are closely associated with the plastic surgeon’s experience. Accurate identification of breast volume is of great importance for patient satisfaction and for the success of the operations performed by the plastic surgeon [1].

Plastic surgeons decide on the breast volume by the rule of thumb, which makes individual experience important [1-3]. Physical methods such as taking a mold and steeping in water are invasive methods that require more time. Volume could be identified by taking the specimen in a cup after the operation according to the method called Golden Proportion of Archimedes' principle, but this would be valid after the operation [4-5]. Digital methods such as three-dimensional (3D) laser scanning, magnetic resonance imaging, ultrasonography, biostereometric analysis and computed tomography (CT) were also utilized. However, answers to the “What are the real measures of breast” question should also be evaluated in terms of anthropometric aspects [5]. Determination of breast shape, size and symmetry by anthropometric measurements is the most scientific method.

Patients apply for breast reduction operations, which are very common today, sometimes with back pain complaints and sometimes with the desire to make changes in the aesthetic appearance. Landmarks (previously identified as anatomic points) that show the success of breast reduction operation are not sufficient. Anthropometric points and their identification are of great importance for choosing the landmarks and identifying the statistical approaches to be used. Except for one or two criteria that are taken into consideration, the success of the operation cannot be evaluated. Patient satisfaction is of importance in these operations; while the decrease in the breast volume decreases patient complaints, the breasts’ being too small could cause psychological trauma. Having perky breasts is of importance for visuality [6]. This study aims to compare breast volume changes and other anthropometric measurements by using before and after breast reduction pictures of women who underwent breast reduction operation in Plastic and Reconstructive Surgery clinic and by performing measurements from the anatomic points indicated in the literature. Targeted patient satisfaction with breast reduction is difficult to standardize. Findings of the present study are believed to contribute to the knowledge about breast reduction, which brings optimum success in terms of health and aesthetics.

## Materials and methods

**Figure 1:**
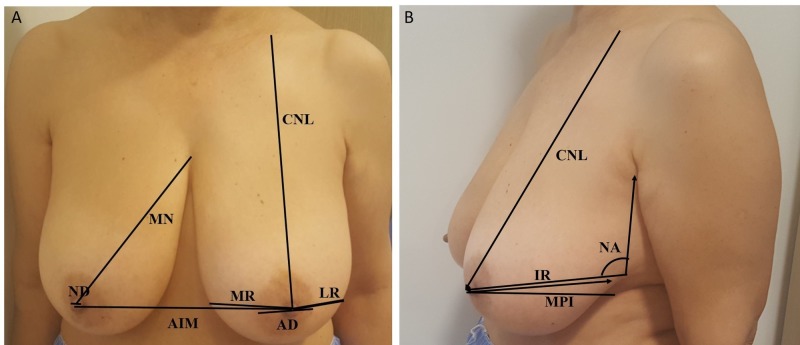
Anatomical points (landmark) in breast CNL: distance between clavicle and nipple, MN: Nipple to medial end point, AIM: distance between two areolas, MR: medial semidiameter of the breast, LR: lateral semidiameter of the breast, IR: Distance between breast-inframammarial sulcus, AD: Areola diameter, ND: Nipple diameter, Breast projection (MP): The distance of the areola mammae from the anterior chest wall of the patient standing in a normal anatomical position, NA: Nipple angle

A total of 40 women whose average ages were 48.3±3.7 years, were measured for breast anthropometric measurements for pre- and post-operative breast reduction surgery changes. All women consist of patients who applied for examination at the Faculty of Medicine Plastic and Reconstructive Surgery Polyclinic. The patients who underwent breast reduction operation by inferior pedicle technique were included in the study. Measurements were taken from the photographs using the Image J programme from the anatomical points (landmark) (Figure 1) [7].

The linear measurements were in millimetre (mm) and were taken photo in the frontal and lateral view by the same author (FO), distance (1.5 meter) and photograph machine in the pre- and post-operative breast reduction surgery.

From the landmarks in literature, 9 double right and left breasts linear distances and 1 linear distance between nipples were calculated and averaged for all women. The difference between the right and left breast were calculated statistically. Reported in the harmonization and agreement between the breast anthropometric measurements changes in the specified regions.

In the present study we used these landmarks: Anthropometric measurements of the breast: CNL: distance between clavicle and nipple, MN: Nipple to medial end point, AIM: distance between two areolas, MR: medial semidiameter of the breast, LR: lateral semidiameter of the breast, IR: Distance between breast-inframammarial sulcus, AD: Areola diameter, ND: Nipple diameter, Breast projection (MP): was defined as the distance of the areola mammae from the anterior chest wall of the patient standing in a normal anatomical position, NA: Nipple angle, areola in case of standing in normal anatomic position (Figure 1), it is defined as the distance of breast to the chest front wall [7].

According to the anatomic (anthropometric) measurement method, breast volume is measured using anatomic dimensions and a geometric volume formula. The most common formula is the one proposed by Qiao et al, [8] as follows:

BV: Breast volume == π/3 × MP^2^ × (MR + LR + IR - MP)

MP = mammary projection, MR = medial breast radius, LR = lateral breast radius, and IR = inferior breast radius. The measurements should be performed when the patient is in a sitting or standing with her arms at her sides.

This study was approved by the Ethics Committee of the Hitit University Clinical Research.

Statistical analysis

Statistical analysis was performed using Statistical Package for the Social Sciences (SPSS) software (Version 22.0, SPSS Inc., Chicago, IL, USA). Normality distribution was assessed using the Kolmogorov Smirnov and Shapiro Wilks tests. For left and right breast anthropometric measurements and breast volume comparisons before and after the operation, paired t-test was used for the data that distributed normally and non-parametric Wilcoxon signed rank test for the data that did not distribute normally. The intraclass correlation coefficient (ICC) using an absolute agreement definition with its 95% confidence interval (CI) and the Bland-Altman plots with the 95% limits of agreement (LoA) (95% LoA=mean difference ± 1.96 SD) were used to determine the agreement between each pair of measurements. The ICC values were interpreted as follows: poor reliability (< 0.5); moderate reliability (0.5-0.75); good reliability (0.75-0.9); excellent reliability (> 0.9). The Bland-Altman Plots were created using “BlandAltmanLeh” package in R (version 3.5.0) software. The value of p<0.05 showed that the study was statistically significant.

## Results

Descriptive statistics and mean comparisons in relation to the parameters measured in the study are presented in Table 1.

**Table 1:** Descriptive statistics for measurements by breast sides (n=40) Statistically significant p<0.05; ^a^: paired t test, ^b^: Wilcoxon signed rank test, CNL: distance between clavicle and nipple, MN: Nipple to medial end point, AIM: distance between areola, MR: medial semidiameter of the breast, LR: lateral semidiameter of the breast, IR: Distance between breast-inframammarial sulcus, AD: Areola diameter, ND: Nipple diameter, MPI: Breast projection, NA: Nipple angle, BV: Breast volume

	Measurements	Before	After	P value
		Mean±SD or Median (min-max)	Mean±SD or Median (min-max)	
Right	CNL (mm)	33.5 (26-45)	21 (21-23)	<0.001^b^*
	MN (mm)	20.50±4.26	15.18±2.40	<0.001^a^*
	MR (mm)	10.7±2.30	11.73±1.71	0.020^a^*
	LR (mm)	7 (4-10)	5 (2-8)	<0.001^b^*
	IR (mm)	22.33±2.73	12.48±2.25	<0.001^a^*
	AD (mm)	6(4-10)	4 (3-5)	<0.001^b^*
	ND (mm)	1 (1-2)	1 (1-2)	0.059^b^
	MPI (mm)	18.03±1.70	14.95±1.99	<0.001^a^*
	NA ° (mm)	103.38±8.54	60.15±5.39	<0.001^a^*
	BV (cm^3^)	7559.5 (4537.3-13677.8)	3477.0 (1657.9-7536.0)	<0.001^b^*
Left	CNL (mm)	33 (26-46)	21 (21-22)	<0.001^b^*
	MN (mm)	20.47±4.07	15.55±2.71	<0.001^a^*
	MR (mm)	10.63±2.33	11.98±1.79	0.004^a^*
	LR (mm)	6 (4-10)	5 (2-8)	<0.001^b^*
	IR (mm)	22.08±2.58	12.50±1.96	<0.001^a^*
	AD (mm)	6(4-10)	4 (3-5)	<0.001^b^*
	ND (mm)	1 (1-2)	1 (1-2)	0.180^b^
	MPI (mm)	18.38±2.13	15.10±2.01	<0.001^a^*
	NA °	103.07±8.09	61.72±5.36	<0.001^a^*
	BV (cm^3^)	6869.8 (3751.3-12664.7)	3581.1 (1768.9-7385.3)	<0.001^b^*
	AIM	27.43±4.54	28.55±4.14	0.052^a^

Right and left breast indicated non-significant differences only between ND before-after measurements (p=0.059, p=0.180, respectively). All the other measurements demonstrated statistically significant differences (p<0.05, Table 1). The distance between areola (AIM) before-after measurements demonstrated no statistically significant differences (p=0.052, Table 1).

Table 2 presents agreement statistics between before and after breast reduction right and left breast measurements (ICC) taken from different points.

**Table 2:** Intraclass Correlation Coefficients (ICC) and confidence intervals (CI) for the different measurements CI: Confidence interval, ICC: Intraclass Correlation Coefficients, MN: Nipple to medial end point, MR: medial semidiameter of the breast, LR: lateral semidiameter of the breast, IR: Distance between breast-inframammarial sulcus, MPI: Breast projection, NA: Nipple angle

	Measurements	N	ICC		P value
			ICC	CI %95	
Before Breast Reduction	MN (R) – MN (L)	40	0.926	0.864 – 0.960	<0.001
	MR (R) – MR (L)	40	0.937	0.884 – 0.966	<0.001
	LR (R) – LR (L)	40	0.620	0.363 – 0.785	<0.001
	IR (R) – IR (L)	40	0.872	0.773 – 0.930	<0.001
	MPI (R) – MPI (L)	40	0.821	0.682 – 0.902	<0.001
	NA (R) - NA (L)	40	0.836	0.710 – 0.910	<0.001
	VOLUME (R) - VOLUME (L)	40	0.954	0.913 – 0.976	<0.001
After Breast Reduction	MN (R) – MN (L)	40	0.850	0.733 – 0.918	<0.001
	MR (R) – MR (L)	40	0.740	0.561 – 0.853	<0.001
	LR (R) – LR (L)	40	0.730	0.537 – 0.849	<0.001
	IR (R) – IR (L)	40	0.857	0.745 – 0.922	<0.001
	MPI (R) – MPI (L)	40	0.969	0.941 – 0.984	<0.001
	NA (R) - NA (L)	40	0.768	0.564 – 0.877	<0.001
	VOLUME (R) - VOLUME (L)	40	0.942	0.891 – 0.969	<0.001
Differences of Before-After Breast Volume	R-L	40	0.932	0.870 – 0.964	<0.001

All the measurements showed statistically significant agreement (p<0.001, Table 2). An analysis of Table 2 shows that MN, MR right and left breast agreement measurements showed excellent reliability level. Agreement between right and left breast measurements in IR, MP and NA areas indicated good reliability level. Only the agreement between right and left breast measurements in LR area was at a moderate level. After the operation, MP right and left breast measurements indicated excellent reliability level. Agreement between MN, IR and NA areas right and left breast measurements was at good reliability level. Agreement between right and left breast measurements in MR and LR area had moderate reliability level (see Table 2). Agreement between right and left breast volumes both before and after the operation were found to have excellent reliability level (p=0.954 (0.913 - 0.976), p=0.942 (0.891-0.969), respectively). The right and left breast volume agreement after the operation was very close to breast volume agreement before the operation. In addition, differences between before and after operation breast volumes were calculated for right and left breasts separately, and agreement between them was found to have excellent reliability level 0.932 (0.870-0.964).

Mean differences and 95% limits of agreement (LoA) of the measurements taken from different areas after breast reduction are demonstrated in Table 3.

**Table 3:** Agreement of methods for different measurements LoA: Limits of Agreement, SD: Standart Deviation, MN: Nipple to medial end point, MR: medial semidiameter of the breast, LR: lateral semidiameter of the breast, IR: Distance between breast-inframammarial sulcus, MPI: Breast projection, NA: Nipple angle

	Measurements	Means of differences			LoA (95%)	
		N	Outside the LoA	Mean±SD	Lower	Upper
After Breast Reduction	MN (R) – MN (L)	40	1	-0.375 ±1.372	-3.063	2.313
	MR (R) – MR (L)	40	1	-0.250 ±1.256	-2.711	2.211
	LR (R) – LR (L)	40	0	0.400 ± 1.150	-1.854	2.654
	IR (R) – IR (L)	40	2	-0.025 ± 1.143	-2.266	2.216
	MPI (R) – MPI (L)	40	1	-0.150 ± 0.483	-1.097	0.797
	NA (R) - NA (L)	40	0	-1.575 ± 3.426	-8.290	5.140
	VOLUME (R) - VOLUME (L)	40	1	-12.04±558.92	-1107.5	1083.4

**Figure 2:**
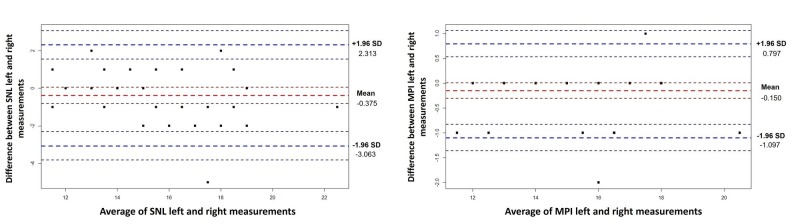
Bland-Altman Plots for MN and MPI measurements The y-axis on the graph represents the measurement differences, while the x-axis represents the average of the measurements. Red dashed lines represent the mean differences with confidence interval and blue dashed lines represents the 95% limits of agreement with lower limit and upper limit. MN: Nipple to medial end point, MPI: Breast projection

**Figure 3:**
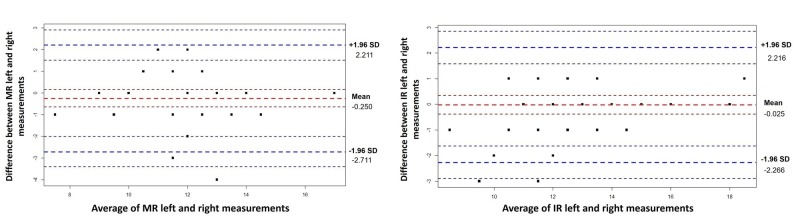
Bland-Altman Plots for MR and IR measurements The y-axis on the graph represents the measurement differences, while the x-axis represents the average of the measurements. Red dashed lines represent the mean differences with confidence interval and blue dashed lines represents the 95% limits of agreement with lower limit and upper limit. MR: medial semidiameter of the breast, IR: Distance between breast-inframammarial sulcus

**Figure 4:**
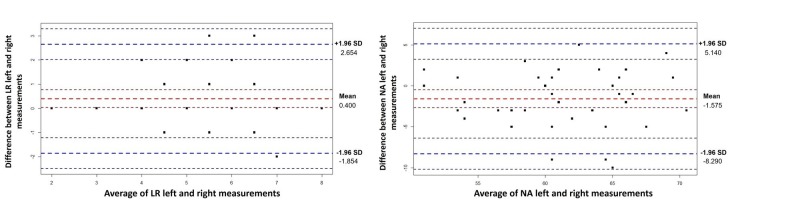
Bland-Altman plots for LR and NA measurements The y-axis on the graph represents the measurement differences, while the x-axis represents the average of the measurements. Red dashed lines represent the mean differences with confidence interval and blue dashed lines represents the 95% limits of agreement with lower limit and upper limit. LR: lateral semidiameter of the breast, NA: Nipple angle

**Figure 5:**
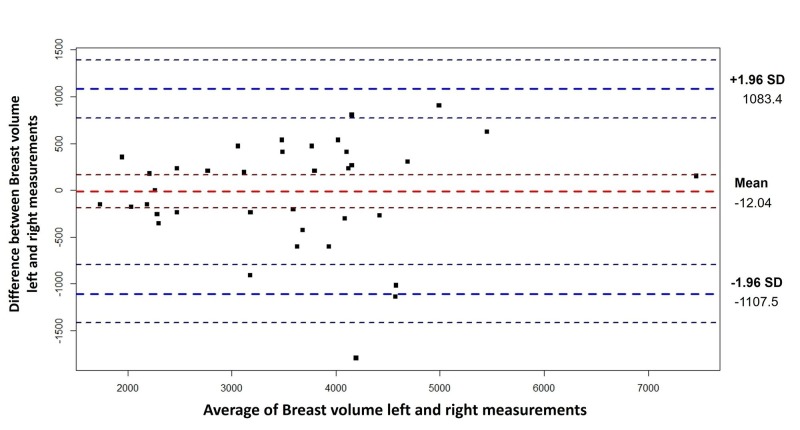
Bland-Altman plots for breast volume left and right measurements after breast reduction The y-axis on the graph represents the measurement differences, while the x-axis represents the average of the measurements. Red dashed lines represent the mean differences with confidence interval and blue dashed lines represents the 95% limits of agreement with lower limit and upper limit.

The Bland-Altman graphics are demonstrated in Figures 2-5. Generally, all measurements of right and left breast after the operation were within levels of agreement according to the measurement areas (Table 3). An analysis of Figures 2-4 shows that 1 measure between MN (R)- MN (L), 1 measure between MP (R)-MP (L), and 2 measures between IR (R)-IR (L) were not within reliability limits.

All measurements between LR (R)-LR (L) and NA (R)-NA (L) were within reliability limits. An analysis of Figure 5 indicates that 1 measure between right and left breast volume measurements was out of reliability limits.

## Discussion

Patients would like to have breast reduction in order to have less social and sexual embarrassment and reach a better quality of life in conducting physical activities and finding appropriate clothes. Women with breast hypertrophy might experience low self-esteem and thus want to have operation in order to decrease the physical and emotional discomfort. Reductive mammoplasty is quite effective for the improvement of the functional, aesthetic and psychological problems, and various studies in literature have demonstrated its effectiveness in increasing quality of life [8-9].

In our study, we compared the breast size of 40 women who had breast reduction surgery before and after the operation. Generally, all measurements of right and left breast after the operation were within levels of agreement according to the measurement areas.

Heavy and big breasts cause neck, back and breast pain due to the pressure of bra straps and maceration at the inframammary area. Breast tissues should be reduced in a way to decrease almost all symptoms related to heavy breasts, without ignoring the blood stream to nipple-areola complex. On the other hand, due to excessive pedicle length particularly in patients with gigantomastia, it is not always possible to preserve blood flow to nipple-areola complex [10]. In this study, we aimed to describe and compare changes in breast volume and other anthropometric measurements in before and after breast reduction surgery.

Keçeci ve Sır (2011) investigated 39 women who participated in breast reduction were included in the study. The nipple to inframammary fold distance (NIMF), MN, lateral end point (LN) for nipple distance, upper limit of the tip of the nipple (SN) of the nipple, breast circumference (BC) and the sternal notch for sealing distance chest circumference (CC) was measured. Keçeci and Sır (2011) showed that the regression method based on anthropometric measurements by using these measurement points was accurately predicted [11]. The mean resection weight was 809 g (standard deviation (SD) 387). The present study involved 40 women whose breast volumes were measured; findings indicated breast volumes of right and left to be 7566±2079 and 7382±2010 before the operation, and right and left to be 3490±1196 and 3502±1167 after the operation.

Zheng et al. (2007) analyzed bare breast measurements from 456 subjects aged 20 to 39 years, who were Chinese women, and tried to offer a new bra sizing proposal [12]. The new sizing system uses the underbust circumference and nozzle depth width ratio as classification parameters. They are defined as two critical parameters by fundamental component factor analysis and K-mean clustering analysis. In addition to 98 measurements from the 3D body scan and other related tits, they also used five additional manual measurements. They also investigated the breast dimensions with basic parameters including distance, width, thickness, volume and curvature.

Our study also took the volume expansion and anthropometric measurements of women from Anatolia population. The difference in the present study is that the participating patients were complaining about the size and heaviness of their breasts; we had the chance to identify the changes by taking the measurements after the operation.

Veitch et al. (2012) investigated breast reduction [13]. Upper and lateral breast tissue was not included in the study. Breast Volume Measurement Using Body Scan Technology Work at the Flinders Medical Center, included mastectomy volume verification experiment. A total of 39 mastectomies of 30 patients were passed through the system. For mastectomy, the sample is weighed and the volume is calculated and this is done in situation. As for the results, the volumes were very high and correlated r = 0.095. The volume of each person's 3D scan volume was slightly higher than the mastectomy volume. This could be because of the inclusion of the skin. In screening volume calculations, researchers proposed a formula for correcting the volume of the deeply derived volume. The present study calculated volumes using anthropometrical measurement methods. It was not used in CT patients due to the probability of causing negative effects on their health.

Kayar et al. (2011) investigated the breast volume of 30 patients, total mastectomy before the surgery was measured [14]. They use five different methods. These are mammography, anatomical (anthropometric), thermoplastic casting, Archimedes procedure and Grossman-Roudner device. The water displacement method (Archimedes) was used to measure the sample volume after total mastectomy in each patient. The results obtained with five different methods were compared statistically with the values. The mean mastectomy specimen volume was 623.5 mL (range 150-1490). The breast volume values were established to be 615.7 mL (r = 0.997) with the mammographic method, 645.4 mL (r = 0.975) with the anthropometric method, 565.8 mL (r = 0.934) with the Grossman-Roudner device, 583.2 mL (r = 0.989) with the Archimedes procedure, and 544.7 mL (r = 0.94). The present study performed volume measurements with anthropometric methods. Breast volume resected after the operation was 4076±1566 ml for right breast and 3880±1466 ml for left breast.

Anthropometric measurements increase the predictability of the result after the operation in plastic surgery practice. However, it is difficult to expect the outcome to be satisfactory for the patient. Geographical and socio-cultural differences change the expectations of patients after surgery. The retrospective reports of the country where surgery is performed are important for estimating the ideal breast when considering social differences. In a study examining breast size of 385 people in Turkish population, the mean breast volume was measured as 407.2 ± 263.6 cc. [15]. In our study, the breast masses obtained after surgery approach to the average of Turkish society may increase patient satisfaction.

The determination of the height and weight of the patients with anthropometric measurements may also allow us to estimate patient satisfaction after surgery. Although some studies in the literature showed that gain in weight increased the breast volume, there was not enough study showing the relationship between weight gain and breast volume [16-17]. One of the limitations of our study is that the height, weight and body mass indexes of the patients were not determined before and after the surgery.

As in previous studies, in our study, we found statistically significant reduction in AD as in breast volume after breast reduction surgery [16]. While AD significantly decreased after surgery, ND remained the same. In a study evaluating the size of the areola, nipple and breast, the average areola: nipple proportion was 3: 1 [18]. In our study, considering the changes in the diameter of areola after surgery; not assessing these rates may be considered as the limitation of the study.

## Conclusions

This study presented anthropometric measurements to show and guide patient satisfaction and aesthetic success of the operations performed by plastic surgeons. Through these measurements, quantitative success of surgeons could be demonstrated. These kind of studies enable plastic surgeons to know the amount of the breast volume (cc) before the operation.
